# Knowledge and perspectives of female genital cutting among the local religious leaders in Erbil governorate, Iraqi Kurdistan region

**DOI:** 10.1186/s12978-018-0459-x

**Published:** 2018-03-07

**Authors:** Hamdia M. Ahmed, Mosleh S. Kareem, Nazar P. Shabila, Barzhang Q. Mzori

**Affiliations:** 1 Department of Community Medicine, Hawler Medical University, Erbil, Kurdistan Region Iraq; 2 College of Nursing, Hawler Medical University, Erbil, Kurdistan Region Iraq; 3 Directorate of Health, Erbil, Kurdistan Region Iraq

**Keywords:** Female genital cutting, Religious leaders, Women sexuality, Culture, Erbil governorate

## Abstract

**Background:**

Religious leaders are one of the key actors in the issue of female genital cutting (FGC) due to the influential position they have in the community and the frequent association of FGC with the religion. This study aimed to assess the knowledge and perspectives of the local religious leaders in Erbil governorate, Iraqi Kurdistan Region about different aspects of FGC.

**Methods:**

In-depth interviews were conducted with a sample of 29 local religious leaders. A semi-structured questionnaire was used that included questions about their knowledge, understanding, and perspectives on different aspects of FGC such as the reasons for practicing it, their contact and communication with the community regarding the practice and perspectives about banning the practice by law.

**Results:**

Participants believed that FGC is useful for reducing or regulating the sexual desire of women to prevent adultery and engagement in pre and extramarital sexual relations and to enhance hygiene of women. They indicated that there is no any risk in doing FGC if there is no excessive cut. Most participants indicated that FGC is attributed to the religion and some considered it a tradition mixed with the religion. People rarely ask the advice of the religious leaders regarding FGC, but they frequently complain about the effects of the practice. Participants did not support having a law to ban FGC either because they thought it would be against the religion’s advice on FGC or it will not work.

**Conclusions:**

The local religious leaders lack adequate knowledge about different aspects of FGC particularly the health consequences. There are different and disputing viewpoints about the reasons for practicing FGC, and there is poor support for having a law banning the practice. There is an essential need for enhancing the knowledge of the local religious leaders regarding FGC and its adverse effects on the women’s health.

**Electronic supplementary material:**

The online version of this article (10.1186/s12978-018-0459-x) contains supplementary material, which is available to authorized users.

## Plain English summary

Female genital cutting (FGC) is the cutting or removal of part or all of the external female genitalia for non-medical reasons. FGC is commonly practiced in the Iraqi Kurdistan Region where the local religious leaders have an important role in the community. The aim of this study was to assess the knowledge and the viewpoints of local religious leaders in Erbil governorate, Iraqi Kurdistan Region of FGC.

In-depth interviews were conducted with 29 local religious leaders in Erbil governorate, Iraqi Kurdistan Region. Questions were asked about their knowledge, understanding, and viewpoints of different aspects of FGC. The local religious leaders believed that FGC is useful for reducing the sexual desire of women to prevent them from pre and extramarital sexual relations. They indicated that FGC would not cause any harm if the cutting is not excessive. Many of them thought that FGC is required by the religion and some considered it a tradition mixed with the religion. People rarely ask the advice of the local religious leaders regarding FGC, but they frequently come to them to complain about its complications. The local religious leaders did not support having a law to ban FGC because they thought it would be against the religion’s advice on FGC.

The local religious leaders lack enough knowledge about FGC particularly the health complications. There is poor support for banning the practice by law. There is an important need for increasing the knowledge of the local religious leaders regarding FGC and its complications.

## Background

Female genital cutting (FGC) is the cutting or removal of part or all of the external female genitalia for non-medical reasons. The World Health Organization classification describes four types of FGC: clitoridectomy, excision, infibulations, and other procedures [1].

It is widely recognized that FGC violates a series of human rights principles. It is also an important manifestation of gender inequality and discrimination [1, 2]. FGC has many serious implications for the health of girls and women. It often causes pain, bleeding, infection, and dysuria as immediate consequences of the procedure. It also causes chronic pain, chronic infections, poor quality of sexual life, birth complications, and psychological problems as long-term effects [1, 3, 4]. More than 125 million women have experienced some types of FGC in 29 countries across Africa and the Middle East while another 30 million girls are at risk of being cut in the next decade [5].

FGC is commonly practiced in the Iraqi Kurdistan region, which is particularly concentrated in Erbil and Sulaymaniyah governorates [5]. The prevalence of FGC in the Iraqi Kurdistan Region is around 40%. However, this prevalence varies by geographical locations from 4.6% in Duhok governorate to 62.9% in Erbil governorate and 55.8% in Sulaymaniyah governorate. The prevalence is close to 100% in some specific rural areas [6]. The most common type of FGC in the Iraqi Kurdistan Region is Type I (76–99%), which includes partial or total removal of the clitoris and/or the prepuce [7, 8].

The roots of FGC in the Iraqi Kurdistan Region are unclear. FGC is prevalent in Iraqi and Iranian Kurdish areas [9, 10] but rare in the rest of Iraq or the Turkish Kurdish area. FGC is deeply rooted in the cultural and social values and beliefs of the affected communities. Social and cultural traditions are considered important reasons for performing FGC in different countries, including the Iraqi Kurdistan Region (40.7% to 46.7%) [7, 8, 11]. Many people consider FGC a beneficial cultural practice and in the best interest of the child [12]. Many parents might subject their girls to FGC thinking that they are “protecting” them from being ostracized and socially excluded from the community [13]. FGC is believed to protect women’s chastity through reducing libido. Therefore, it is usually associated with the cultural principles of modesty and femininity [12, 14].

In the Iraqi Kurdistan Region, the Domestic Violence Bill that was passed in June 2011 includes several provisions criminalizing female genital mutilation in Kurdistan. The bill listed female genital mutilation among13 items of domestic violence. The bill sets penalties for encouraging and performing female genital mutilation practice with a fine, imprisonment and banning the health professionals from the practice [15]. The regional government also established the High Council of Women Affairs, a governmental agency directly linked to the Prime Minister’s office and responsible for combatting all types of gender-based violence including FGM. Several civil society organizations are also actively involved in these efforts to reduce FGC practice [8].

Various reasons are given for practicing FGC in different communities. However, the practice has been linked to Islam in the predominantly Muslim communities, and there is a strong belief that every Muslim woman must be subjected to FGC [16]. Religious obligation or requirement is an important reason (38.8% to 50.3%) for practicing FGC in the Iraqi Kurdistan Region [7, 8]. In fact, FGC is not an Islamic problem and it is practiced in many non-Muslim communities. The practice predates Islam and there are many majority Muslim countries where the incidence of FGC is very limited [17]. The presence of religious scripts that explicitly prescribe or encourage FGC is usually denied in the literature. Some renowned Sunni and Shi’i Islamic scholars, including a scholar from the Iraqi Kurdistan region have dismissed any association between FGC and Islam and even issued fatwa^1^ forbidding FGC [18–20]. However, many people still believe that FGC has religious support [21] and in some countries arguments inspired by Islamic law have been used to claim that FGC is an obligation in Islam (17). In the Iraqi Kurdistan Region, girls and women who are not cut might be considered to have *haram*^2^ hands, and some people do not eat or drink from their hands [11]. These unexamined aspects of FGC might play a crucial role in the high prevalence of FGC in the Iraqi Kurdistan Region.

Understanding the views of different actors in the community about FGC is very important to uncover the motivations behind the practice and ensure the effectiveness of preventive programs. The limited research from the Iraqi Kurdistan Region has primarily assessed the prevalence of FGC and its associated factors. Research has rarely examined in an in-depth manner the knowledge and perspectives of the influential people in the community, such as the local religious leaders of this practice and their potential role in combating this harmful practice.

Stopping FGC needs to start at the grassroots level through the participation of all the key players in the community, such as religious leaders, advocates, and educators [22]. Religious leaders are one of the key actors in the issue of female genital cutting (FGC) due to the important position they have in the community and the frequent association of FGC with the religion. It has been suggested that religious leaders have a substantial influence on whether the practice persist or not [17]. Moreover, several programs show that rapid elimination of FGM can be achieved if communities, supported by religious leaders, decide to abandon the practice [23, 24]. Some reputed Islamic scholars in the Iraqi Kurdistan Region have publicly condemned the practice of FGC, while others preferred to stay silent or even encouraged the practice [20]. Therefore, this study aimed to assess the knowledge and perspectives of the local religious leaders in Erbil governorate, Iraqi Kurdistan Region about different aspects of FGC.

## Methods

This interview-based qualitative study was conducted in Erbil governorate, Iraqi Kurdistan Region, from June 2016 to May 2017.

We selected a purposive sample of 40 local religious leaders to represent different geographical areas of Erbil governorate including both urban and rural areas, areas of the different socioeconomic conditions and areas with varying prevalence of FGC. The sample included both imams^3^ and preachers (*khateebs*)^4^ from the mosques and Islamic academic scholars from the College of Islamic Sciences and of different educational levels. We identified the participants by consulting two key contacts; an Imam and a scholar. One of the authors contacted the potential participants by phone and invited them to take part in the interviews.

A semi-structured questionnaire was developed and used to guide the in-depth interviews (Additional file 1). The questionnaire included questions about the demographic and professional characteristics of the participants. It also included questions about the knowledge, understanding, and perspectives of the local religious leaders of different aspects of FGC including the definition, types, performers, reasons for practicing it and the prevalence in the Kurdish community in addition to their contact and communication with the community and their perspectives about banning FGC by law. We purposely did not ask about their knowledge of the existing domestic violence law that also criminalizes FGC in the Iraqi Kurdistan Region not to influence their answers about their views on banning the practice by law. The semi-structured questionnaire was pre-tested to determine the accuracy and the understanding of the questions. Two male surveyors interviewed the participants, and each interview lasted around one hour.

The study was approved by the Research Ethics Committee at Hawler Medical University. The participants were informed about the purpose, and the importance of the study and the informed consent was obtained before the interview. The anonymity of the participants was ensured throughout the different stages of the study.

Three researchers separately conducted the interviews (MSK; 12 interviews, BQM; 12 interviews and HMA; 5 interviews). All interviews were conducted in the Kurdish language. Most interviews were entirely audio recorded. For the few participants who did not agree to record their interviews, full notes were taken by the interviewer. The recordings were transcribed before being translated into the English language. The translation was verified by a native Kurdish speaker who was fluent in English. We used content analysis to analyze the translated transcripts qualitatively. This type of analysis aimed to approach the study topic without any preconceived ideas to allow new perceptions based on the collected data. The transcripts were reviewed by two authors independently. They compared their notes and reconciled any differences. The condensed meaning units were identified and summarized. Then, they were abstracted and labeled with codes. The emerging codes were used to obtain categories. The two authors further discussed these categories for identification and formulation of themes. A greater emphasis was placed on the themes repeated by more than one participant, themes of long discussions or strong feelings. The discordant views were included to underline differing knowledge or perceptions of the study participants.

The four criteria of transferability, credibility, dependability, and conformability were used to ensure the rigor of the study. This involved presenting sufficient quotations in the results section, using a semi-structured interview guide, checking some of the coded interviews by academic staff and conducting the preliminary analysis by two authors with the third author independently reviewing the process.

## Results

Of the 40 local religious leaders, 29 agreed to participate in the study. The mean ± SD age of the participants was 48.9 ± 14.9 and ranged between 32 and 76 years. Twenty participants were Imams in mosques; 13 were from Erbil city center, and seven were from the towns and rural areas located within 60 km of Erbil city. The other 9 participants were Islamic academic scholars teaching at the College of Islamic Sciences in Erbil city. Four participants had a scientific certification to practice, eight had a diploma degree, seven had a bachelor degree, six had a master degree, and four had a Ph.D. degree. The 29 interviews provided a wide representation of views and sufficient saturation. The results have been categorized into the following seven categories: understanding of FGC, advantages and disadvantages of FGC, reasons for practicing FGC, contact with the community regarding FGC, the prevalence of FGC in the Iraqi Kurdistan Region, the geographical difference in the prevalence of FGC and banning FGC by law as shown in Table 1.

**Table 1:** The main categories and sub-categories of the results extracted from the interviews

Category	Sub-category	Participant’s number with relevant quotation
Understanding FGC	Definition	15; 23
	Type	15; 22
	To whom	10; 17; 19; 23
	By whom	16
	Role of *sharia*^a^	5; 10; 19
Advantages and disadvantages	Reducing sexual desire	5; 23
	Better hygiene, reduced smell	5; 21
	No risk if well done	All
	Insufficient desire and pleasure	5; 16
Reasons for practicing FGC	The prophet said it	17
	In the *sharia*	27
	Culture NOT religion	20; 21; 28
	Regulating sexual activity	13; 15; 23
	Smell	5; 28
Contact of religious leader with the community regarding FGC	The families do not ask	14; 15; 16; 18; 24
	Complaints from men re sex	3; 5
	Hemorrhage post FGC	13
	Shame and right to complain	16; 17
	Unfaithful if no FGC	19
Prevalence of FGC in Iraqi Kurdistan	Decreasing	13; 21
	Unknown because secret for girls	23
Geographical differences inside and outside Iraqi Kurdistan	Shafi^b^ vs. Hanafi^c^ doctrine	5; 10; 16; 22
	Faith	5; 15
	Education	18; 21
	Weather/heat	5; 22
	Tradition	16
	Unclear	1
	Pressure groups	23
Banning FGC by law	Knowledge of the existing law	22
	Do not support	9; 12; 17; 29
	Call for expert opinion	13; 14

^a^*Sharia* is the religious law forming part of the Islamic tradition

^b^Shafi’i doctrine is one of the four schools of Islamic law in Sunni Islam. The Shafi school predominantly relies on the Holy Quran and the *hadiths* for *sharia*. Where passages of the Holy Quran and hadiths are ambiguous, the school first seeks religious law guidance from Ijma – the consensus of Sahabah (Prophet Muhammad’s companions). If there was no consensus, the Shafi’i school relies on the individual opinion (*Ijtihad*) of the companions of Prophet Muhammad (peace be upon him), followed by analogy

^c^Hanafi doctrine is one of the four religious Sunni Islamic schools of jurisprudence. Hanafi doctrine derives Islamic law from the Holy Quran, and the *hadiths* containing the words, actions, and customs of the Prophet Muhammad (peace be upon him)

### Understanding of FGC

The local name of circumcision (*khatana*), which is derived from the Arabic name, was primarily used by the participants during the interviews. However, some participants referred to other names used in religion such as “*khafedh*” which means to mitigate or reduce.

FGC was mostly defined as cutting off a part of the clitoris. Clitoris sometimes was called by its correct Kurdish name (*mitka*) or Arabic name (*bazr*), but most of the times a simulated name was used such as “the extra piece of meat at the upper part of the vulva,” or it was simulated to “the chicken or rooster caruncle.”

“As *sharia* says, this is from *the fiqh*^5^ book, circumcision in women is cutting the furthest part of skin at the upper vulva.” (Interview 23).

“Clitoris has its location in the upper part of the vulva of women. It becomes straight. Doctors should decide to cut or not and how much to cut. They do not cut more than the limit that damages it. So if it is required, they will only cut it and not anything below it or the side of it. The skin is like a caruncle of chicken or rooster that has a pile.” (Interview 15).

“It is called reducing, which means dropping. It means reducing the heat of a woman's body, the heat of desire. When it is reduced a bit, the heat will not overcome the mind. Heat in *fiqh* means desire.” (Interview 23).

Only one type of FGC was described by most participants, which was the cutting part of the clitoris. Sometimes reference was also made to the more extreme types that are not practiced in the Iraqi Kurdistan Region and in Islam, such as the Pharaonic circumcision that is practiced in Sudan and Egypt.

“There is only one type similar to men’s circumcision. In men is the skin (foreskin) and in women is the clitoris.” (Interview 15).

“There is light circumcision, let's say, and it is clear as it is cutting a part of the clitoris and is cut slightly. There is also a type that is done in Egypt and Sudan, which is called Pharaoh’s circumcision. They exaggerate in cutting.” (Interview 22).

There were different views about to whom FGC is usually performed in the Iraqi Kurdistan Region. Some participants indicated that FGC is needed for those having very large clitoris above the sides (labia) making the region ugly and it may be regarded as a congenital anomaly and very sensitive to sexual desire. They thought that if the clitoris is lower than the edges it does not need cutting. They stressed that medical professionals should decide this issue.

Some participants described the clitoris to be higher in some women, above the two edges of labia causing problems when erected such as annoyance to the husband during sex, and thus it requires cutting. They thought that the cut is according to the need and doctors knows to cut or not and how much to cut.

“Cutting is required when the clitoris is much higher than the sides and makes the area ugly and become very sensitive. I do not know about medical aspect, but it might become something annoying for the woman or is more enjoyable or not for the man; we do not know this. So if it is not large and is lower than the edges, it does not need cutting. *Fuqaha*^6^ (Islamic jurists) and those who say FGC is *Sunnah*,^7^ say if the clitoris is not higher it does not need cutting. If it is small, the cut might harm or take away the sexual pleasure.” (Interview 19).

“Having this extra meat makes sex more enjoyable than not having it. This extra meat is different in some women as it is large and very high. This makes the organ of woman ugly. When the woman grows, the woman becomes annoyed by this organ during sex and many other things such as psychological problems. In some women, it is not very high and is not protruded outside, and it might not be seen at all.” (Interview 19).

Another view was that FGC is practiced in areas of warm climate due to early maturity of girls and increased sexual desire.

“As *sharia* says and from reading the Fiqh books, the sexual desire of women is higher in the regions with the warmer climate, and always the sexual desire is stronger in the warmer areas. I think this is also correct medically. These areas are different from the colder areas.” (Interview 23).

Another view was that FGC is to be done only for women with high sexual desire and at risk of experiencing adultery.

“It is good to be done, but only for the women with high sexual desire. If you know she will indeed experience adultery, it is better to be done, but with her or her parents' permission.” (Interview 10).

The last view was that FGC should be done for all girls and women.

“It needs to be done to every girl since one does not know who has a high sexual desire and who has not.” (Interview 17).

Participants agreed that FGC is mainly practiced by traditional birth attendants in the Iraqi Kurdistan Region, but they stressed that it needs to be performed by female medical professionals or at least experienced traditional birth attendants to avoid complications.

“It was done by traditional birth attendants in the past. A woman was doing it. In my opinion, this is not good today in this situation. I do not like it. I prefer and if possible the people who are specialists in this field to do it. I prefer to be doctors and be a specialist in this area to do the work beautifully and not harming the woman.” (Interview 16).

Participants who were the proponent of FGC argued that the *sharia* has already decided on it and it should be done to all girls. Other participants indicated that the parents need to decide on it and there is a need to have the father’s consent, which is usually not done in the Iraqi Kurdistan Region. Others indicated that doctors should decide if it is required or not.

“*Sharia* decides, but there are different opinions on FGC among the different doctrines (madhabs).” (Interview 5).

“The decision is not by religion, but by a doctor and family.” (Interview 10).

“In my opinion, parents should go to a doctor to examine the girl to see if it is the type that needs circumcision or not.” (Interview 19).

### Advantages and disadvantages of FGC

The participants mentioned different benefits of FGC and the primary focus was on reducing or regulating the excessive sexual desire that women have and the associated deviation, sin and community and social problems. Other advantages mentioned by the participants included enhancing the hygiene and cleanliness of the woman and avoiding the annoyance of the husband during sex.

“Women have a higher sexual desire than men. Cutting this piece of meat in women does not do anything harmful to women. We are a human being, and anything at its limit is a benefit. Cutting part of this piece of meat (clitoris) is for the benefit of the woman to curb this desire. Otherwise, it is possible that women's desire would be that high that she makes sin and exceed her limit.” (Interview 23).

“It regulates or limits the sexuality and the desire of women.” (Interview 5).

“FGC leads to cleanliness and hygiene of reproductive organ of the woman. It removes bad odor that many times happen due to some secretions that affect the woman. In the two small lips (labia minora) some secretions lead to bad odor in the woman organ and infection of the urinary tract.” (Interview 5).

“From the medical aspect, we have many studies that say FGC has some benefits including taking away the odor, will not become a cause of annoyance for men during sex because in some women it is very long and reaches 3 cm.” (Interview 21).

Most participants indicated that there is no any risk in doing FGC while some participants mentioned some disadvantages that only occur if there is excessive cut such as reduced or loss of sexual desire.

“It has harmful effects if it is done in a non-scientific way or if the organ (clitoris) is cut extensively that the woman loses all sexual feeling. This leads to a disaster of marital separation and results in many problems.” (Interview 5).

“If a lot is cut, the first thing from the *sharia* aspect it, harms the body of the woman and harms the sex by losing sexual desire and pleasure. This woman will have less feeling, and when she gets married in the future, she might have problems.” (Interview 16).

### Reasons for practicing FGC

Most participants indicated that the practice of FGC is primarily attributed to the religion, while others considered it a tradition related to culture or a tradition mixed with the religion.

“FGC is related to the religion because the Prophet (peace be upon him) has said it. Even if it was practiced in the old times, it has come into the Islam *sharia* and became part of the *sharia*.” (Interview 17).

“They say it is in *sharia* and it was done in the past. The tradition is mixed with religion.” (Interview 27).

“It is primarily related to the culture, but people think it is related to the religion and apply it.” (Interview 28).

“It is a tradition and cultural practice. In my belief, it has no relation with *sharia*. As I am aware, in the village where FGC was practiced, a preacher has mentioned in *khutba*^8^ several times that FGC is risky and warned people of not disabling their children, but the people did not listen to him, and this is wrong.” (Interview 20).

“In my opinion traditions and customs have their effect in the Iraqi Kurdistan Region. People do the work more due to being a tradition and custom. For example, they do not know religious scripts whether it is allowed or not, has sharia said that or not, but they depend on the daily behavior.” (Interview 21).

Other reasons for practicing FGC that were frequently mentioned by the participants included reducing the sexual desire and regulating the sexuality of women as they considered women to have a high sexual desire by nature. Few participants said that FGC is practiced to enhance the hygiene of women as the women who are not cut might have a bad smell or stinking.

“The wisdom of doing FGC is to refine women’s sexual feeling, which means to reduce the high sexual desire in women and decrease the mechanism leading to desire.” (Interview 13).

“For women, mainly because they have an excessive desire and FGC reduces this desire a bit. It does not take away the desire, but will only reduce it.” (Interview 15).

“It is called reducing, which means dropping. It means reducing the heat of a woman's body, the heat of desire. It does not mean weakening the desire. FGC will reduce the heat a bit, so it will not overcome the mind.” (Interview 23).

“In the two small lips (labia minora), some secretions lead to bad odor in the woman's organ and infection in the urinary tract. This FGC takes away this type of allergy in the woman's reproductive organ. FGC leads to cleanliness and hygiene of the reproductive organ of the woman and removes the bad odor that many times happen due to a group of secretions that affect the woman.” (Interview 5).

“I do not support this (FGC) to be done if it is related to sexual desire. However, if it is related to other things such as leading to annoyance or harm during sex or if there is odor or related to a disease, it can be done. If a woman is chaste and has strong faith, she can control her desire.” (Interview 28).

### Contact with the community regarding FGC

Most participants indicated that nobody has come to ask them for advice on FGC. Some stated that few people have come to ask and these included both men and women, poor and rich, and educated and uneducated. In past times when some of the participants were working in the rural areas, people used to come to ask for their advice on FGC.

“Until now, few people have come to ask questions about FGC; whether to do it or not, is it a *Sunnah* or not. They say that some people tell them to do it and some others say do not do it and they do not have a clear answer about it.” (Interview 14).

“In this area, till now 2-3 women have contacted me asking whether to do it or not. I think it is something that has become less common.” (Interview 18).

“Nowadays, very few people come to ask me. In the past when I was working in a village, many people used to come to ask me “should we circumcise this girl or not?” I was saying circumcise her. There was a woman in our village knowing to perform the practice like a doctor.” (Interview 15).

A participant indicated that people are now asking more about the topic as the media started to talk about banning it.

“The rate of asking in this situation is more than the past. In the past, it was less; now people ask more about this topic because this issue is now raised more frequently in the community and even in media.” (Interview 16).

A participant indicated that these sensitive things are usually not discussed among men because of shame.

“Actually, these things are less discussed among men, as it is considered a shame. Sometimes there were complaints and have been mentioned to me.” (Interview 24).

Most participants indicated that they had received people complaining of FGC particularly husbands complaining of loss of sexual desire of their wives and even asking if they can remarry because of that. Few examples of other complications such as bleeding were also mentioned. Some participants said that some people might not complain directly from the effect of FGC, but when they are asked about the causes of other problems such as divorce, they exclusively refer to the lack of sexual desire in women that is resulted from FGC.

“People complain a lot that their wives are circumcised, and their sexuality is zero. The circumcised woman always feels shortage in front of man regarding sex.” (Interview 3).

“Yes, Muslims complain from the FGC, and the reason is mainly related to performing this practice by unskilled people. The complaint basically leads to marital separation, cheating, and many family problems.” (Interview 5).

“Yes, a woman was talking about her young daughter who had excessive cut and bleeding for many days. The performer has either cut a lot or did not know how to cut it.” (Interview 13).

Participants indicated that many people do not come to talk to them due to the sensitivity of the topic of FGC and feeling ashamed to talk about it.

“Yes, many people have come to ask. They are ashamed to ask or ask very shamefully because it is a sensitive topic. From the *sharia* aspect, nothing should be ashamed of and be asked about, but there is some shame on this issue.” (Interview 16).

There were some extreme views that nobody should complain about this issue because it is the *sharia* issue.

“Nobody has complained, but they have no rights to complain because this is a right of Muslim and Muslims should be committed with *sharia*. *Sharia* says it is an obligation (*wajib*)^9^ for some and *Sunnah* for some. Those who do it, it is a reserve to avoid sin and those who do not do it and consider it a *Sunnah*. God’s willing, they will not be sinners. Nobody has a right on another because the Muslim should be committed to the *sharia*.” (Interview 17).

One participant talked about complaints of not having done FGC and leading to extramarital sex.

“A woman called me asking for advice. She had a number of daughters, and all were circumcised except one of them. All the daughters were grown up based on religion and shame, and all got married. She said that the non-circumcised daughter was cheating on her husband (extramarital sexual relationship). So I immediately related this cheating to the lack of circumcision.” (Interview 19).

### Prevalence of FGC in the Iraqi Kurdistan region

There was some general agreement among the participants that FGC was common in the region in the past, particularly in rural areas and it is much reduced nowadays. It was affected by the role of particular Imams in the community in the past, and it is decreased as education and awareness of people is improved.

“In my opinion, FGC is decreasing gradually in the Iraqi Kurdistan Region, and this is attributed to the people’ education (awareness). It was mainly done in nomads and tribes because these communities lacked an adequate number of scholars. A person is an enemy of what he does not know.” (Interview 13).

“It is not done in the area where I live in Erbil. However, it was practiced in my father’s and my in-law’s families who are from Rawanduz district. It was done for the children, but after the situation has changed and the education level is increased, they also stopped doing it.” (Interview 21).

Some participants argued that it is hard to know how common the practice is since it is mainly done in secret for girls, not like boys.

“It is possible, but it is not clear because usually, FGC is practiced in secret. It is different for boys because this is the tradition and culture of Kurds. In the past, even in the Islamic *sharia*, boy's circumcision was associated with celebration, but for the girl, it is usually hidden. This was done in the villages in the past.” (Interview 23).

### Geographical difference in the prevalence of FGC

Participants did not have adequate knowledge about the observed difference in the prevalence of FGC in the different governorates and areas of the Iraqi Kurdistan Region or by countries. When they were told about that and asked about the reasons for such difference, they provided different reasons for that. Some attributed that to the difference in the religious doctrines as they thought that Erbil and Sulaymaniyah people follow Shafi’i doctrine that considers FGC an obligation while those in Duhok follow the Hanafi doctrine that considers FGC a *Sunnah*. They also attributed the difference in the prevalence among the countries largely to the difference in doctrine. Others attributed it to the difference in people’s commitment to the Shafi’i doctrine and the faith.

“The reason for the high prevalence of FGC in the Iraqi Kurdistan Region and Egypt is related to being on Shafi’i doctrine. In the Shafi’i doctrine, FGC is an obligation for both men and women.” (Interview 5).

“Because Imam Shafi’i considered it an obligation and the people in these places (Erbil and Sulaymaniyah) follow Shafi'i doctrine, but Duhok people follow another doctrine.” (Interview 10).

“Doctrines have a great role, particularly in these places that we mentioned like the Iraqi Kurdistan Region because Imam Shafi'i had a particular opinion, which was different from that of other scholars such as Imam Abu Hanifa, Imam Ahmadi, and Imam Maliki. For Imam Shafi'i, it is an obligation for women and men both.” (Interview 16).

“The difference is due to not taking the order as decisive, not like the Holly Quran script that is decisive. Those who have complete satisfaction with Shafi'i, take it as cutting off and consider FGC an obligation.” (Interview 22).

“From religion aspect, the people in Erbil and Sulaymaniyah are more religious and have more commitment to doctrines than Duhok people.” (Interview 5).

“Not practicing FGC is related to the weak faith. People in some regions or countries have weak faith. I have lived with some of them, and they do not fast or pray until becoming old age. So if the people do not fast or pray, how do they practice FGC?” (Interview 15).

Other participants attributed this difference to the level of the education and awareness of the people in the different regions, particularly with the effect of the awareness campaigns against FGC. They generally thought that FGC is less prevalent in the people having a higher education and awareness level.

“There is a group of things, including the violence against women program, improved education, and increased awareness about family relations problems and sexual weakness of women in the future. The people are not ready to do this harm to their girls any longer.” (Interview 21).

“It is related to the educational aspect of the region. For example, the people of Sulaymaniyah city read more and see more, but some people in the rural area and other places in the Iraqi Kurdistan Region are less educated. The people have heard that this is a *Sunnah* and many people have done it.” (Interview 18).

Some participants explained the difference by the difference in the weather in the different regions.

"The weather has a significant role in this procedure. Duhok is colder than the other areas." (Interview 5).

“In the areas where FGC is practiced, it mainly goes back to customs. In Sudan and Egypt, the area is warm, and FGC is practiced there. It is even practiced in the German area (south of Sulaymaniyah and east of Kirkuk) which I think is warm.” (Interview 22).

Some other participants attributed this difference to the tradition or could not find a reason for that.

“FGC has remained in these places as a tradition.” (Interview 16).

“The answer is very difficult, as not everybody talks about it, and there is no special medical facility that people can visit to have accurate statistics. Therefore, I cannot decide on it. This difference is not clear to me.” (Interview 1).

Other participants argued that the human and women’s rights groups have done a bad thing by raising this issue of FGC.

“It depends on the explanation by scholars and religious Imams and preachers. We actually must follow the *sharia*. In the past, we did not have any dispute on this. Now the women's rights say clearly that it is a *haram* thing done to women. What they say I think is not *sharia* and not from the women's rights aspect because who have created a woman and made a plan for her knows better than you and me. How the God will violate the rights of a woman from the desire aspect.” (Interview 23).

### Banning FGC by law

The participants were not asked about their knowledge of the presence of the already existing domestic violence bill that criminalizes FGC in the Iraqi Kurdistan Region. However, only one participant stated that he is aware of the existence of such a law when the participants were asked about their opinion of banning FGC by law.

“FGC is banned by the domestic violence law. When I was writing my Ph.D. thesis, I referred to that project that was submitted to the parliament for discussion at that time.” (Interview 22).

Most participants did not support having a law to ban FGC because they thought that it would be against the *sharia* and religious advice on FGC or it will not work. Some participants argued that having a law banning FGC will become something suspicious and people will oppose it.

“I do not support banning FGC by law.” (Interview 9).

“I do not agree with banning FGC by the law because a law cannot ban it.” (Interview 12).

“It is not good to ban FGC by law. It is better to have it optional and everybody to do what they like.” (Interview 17).

“The families who frequently practice FGC, for example, those in Garmian area, even if there are a hundred laws, if they are not convinced of it, they do not follow it. But if they have religious Imams and preachers with them, it will for sure have an effect.” (Interview 29).

Other participants argued that there is a need to have a law to regulate the practice based on advice from religious and medical experts either to practice it or not. Some participants even suggested establishing a committee including the local religious leaders to prepare effective legislation about FGC that could be acceptable by the people.

“There should be a law issued and the general *fatwa* committee in the Iraqi Kurdistan Region with experts in this field to sit together in addition to women’s organizations and report it in the media. The media should have a role. If it is a good thing, all people let do it. If it is not good, people will know about it. Now there is some knowledge about it, and it is generally less commonly practiced.” (Interview 13).

“I support to be regulated by law and not prohibited; to be regulated by the law so not everybody can perform this procedure. But to be regulated by the law, from the medical and psychological aspects to consider the person who performs it and the one who is performed for.” (Interview 14).

## Discussion

The participants had a good understanding of the definition of FGC. However, their definition and description were mainly focused on type I FGC. This might be related to the fact that type I FGC is the most common type practiced in the Iraqi Kurdistan Region [7, 8] and today’s position of some Islamic scholars urges Muslims practicing FGC to adopt the most moderate form of FGC [25].

There was a consistent emphasis among the participants on the necessity of FGC for the girls and women in areas of the warmer climate. The participants even related the high prevalence of FGC in warmer climate areas of the Iraqi Kurdistan Region such as Garmian area to that. It is often believed that women become sexually mature earlier in the warmer climate and their sexual desire/arousal is higher than those in the colder climate. This belief is sometimes related to the Islamic religion since early Islam started in the warm climate area of Saudi Arabia and child marriage was common in the pre-Islam time in that region. However, there is no clear evidence to prove this association of early sexual maturity to the warmer climate [26], and we believe that there is no any association between the women’s sexuality and the climatic condition.

The participants believed that the women with larger clitoris need FGC due to having a higher sexual desire. However, there is no clear proof that women with larger clitoris will have a higher sexual desire. Even a study revealed that sexual function was improved in women with a smaller-sized clitoris [27].

The traditional birth attendants are primarily responsible for performing FGC in the Iraqi Kurdistan Region [7, 8] and the participants were aware of this fact. Although they emphasized the need for the medicalization of FGC, we believe that this will not reduce the long-term complications of FGC, has no any benefits and violates the code of medical ethics. It can even result in a setback in the efforts to ban this harmful practice [28].

The participants mentioned different benefits of FGC and the primary focus was on the claimed reducing or regulating the excessive sexual desire to prevent adultery and engagement in pre and extramarital sexual relations. However, FGC clearly violates the rights of women since women have the right to have sexual health and to feel sexual pleasure for the full psychophysical well-being of the person [29]. Moreover, it has been shown that women with FGC do not significantly differ from those without FGC in the mean sexual desire score. Other advantages of FGC mentioned by the participants included enhancing hygiene and cleanliness. FGC is seen as ensuring the hygiene of the female genitalia, which in their natural form are wrongly classified as unclean. It is believed that the girl who is not circumcised has a bad odor because she is not clean and even some people consider the food she prepares *haram* [11, 25]. In fact, FGC has no any proved benefits, and the serious health and psychosocial consequences surpass any claimed benefits.

Most participants indicated that there is no any risk in doing FGC while some participants mentioned some disadvantages that only occur if there is excessive cut such as reduced or loss of sexual desire. However, there is clear evidence that all forms of FGC, including type I, can cause a high rate of complications [30]. The other common complications of FGC include excessive bleeding, delay in or incomplete healing, and tenderness in addition to reduced libido and psychological problems in long terms [3, 7, 8]. The high prevalence rate of FGC and the proportion of medical complications show that FGC is a matter of public health concern. Girls who undergo FGC before ten years of age, which is the case in the Iraqi Kurdistan Region, seem to be more vulnerable to serious complications than those who are older at the time of FGC [3].

Most participants indicated that the practice of FGC is attributed to the religion and some considered it a tradition mixed with the religion. In many settings, an important contribution to the practice of FGC is a religious obligation [31, 32]. Dictate of religion is an important reason (38.8% to 50.3%) for practicing FGC in the Iraqi Kurdistan Region [7, 8]. The presence of religious scripts that explicitly prescribe or encourage FGC is usually denied in the literature [20]. However, FGC and circumcision, in general, have been mentioned in some *hadiths* and some scholars argue that it is at least permissible in Islam as the Prophet (peace be upon him) has not prohibited it. Many people still believe that FGC has religious support, particularly in Islam [21]. Concerning Islam’s view about FGC, some readings of the *hadith* suggest that Islam requires FGC and in some countries arguments inspired by Islamic law have been used to suggest that prohibiting FGC could be un-Islamic [17]. However, this interpretation is questioned by some religious scholars who disagree about whether Islam requires, encourages, permits, or discourages the practice [21]. We strongly believe that there is no any association between Islam religion and FGC. In fact, there is not a single verse in the Holy Quran that can be used as a basis for FGC. However, it includes many verses that condemn any practice that harms the human being. Moreover, the tradition from the *Sunnah* of the Prophet Muhammad (peace be upon him) in support of FGC is not authentic [16]. FGC is practiced in many non-Muslim communities, and the practice predates Islam. Besides, there are many countries of Muslim majority, including those following the Shafi’i school where FGC is not practiced at all [17]. Some renowned Islamic figures have denied any association between FGC and Islam. For example, Sheikh Ali Gomaa, formerly the Grand Mufti of Egypt and then Sheikh Al-Azhar, issued a fatwa forbidding and criminalizing FGC since there is a medical consensus on the harm caused by the procedure [18]. A similar fatwa was also issued by Mohammad Hussein Fadlallah, the Shia Grand Ayatullah of Lebanon [19]. Professor Mustafa Zalmi, a renowned Islamic academic scholar from the Iraqi Kurdistan Region, denied any association between FGC and Islamic religion and that the Holy Quran clearly forbids any harmful action or action that its harm is more than its benefits such as FGC [20]. The High Committee for Issuing Fatwas in Iraqi Kurdistan Region issued a fatwa about FGC in 2010, which indicated that the practice is not prescribed in Islam, but predates it. The fatwa does not absolutely prohibit FGC as it says parents may opt to circumcise their daughters, but it is better to avoid the practice because of the negative health consequences [33, 34].

Although almost all Muslims in the Iraqi Kurdistan Region follow the Shafi’i school, which considers FGC an obligation, not all of them practice FGC. While FGC has roots in the Islamic religion as indicated by many religious leaders, the survival and the continuation of the practice in some areas of the Iraqi Kurdistan Region and near complete lack of the practice in other areas indicate that FGC has primarily become a cultural tradition. Many Muslims of the Shafi’i doctrine in the Iraqi Kurdistan Region do not practice FGC at all, and many have not even heard about it. Although people might be aware of the disadvantages of FGC, they cannot abandon the practice due to cultural beliefs and social pressure. Therefore, the culture plays an important role in practicing FGC in the Iraqi Kurdistan context. Social and cultural traditions are considered important reasons for performing FGC in different countries, including the Iraqi Kurdistan Region (40.7% to 46.7%) [7, 8, 11]. Many people consider FGC a beneficial cultural practice. Parents might subject their girls to FGC thinking that they are “protecting” them from being ostracized and socially excluded from the community [13]. By a careful examination of all aspects of the problem, it is clear that FGC is not an Islamic problem and the practice can only be regarded as a cultural practice rather than a religious one.

Having nobody or few people asking the advice of Imams and preachers about FGC might indicate that their role in the practice might not be so important. Some participants indicated that people approached them more when they were working in rural areas. This fact, in addition to the high prevalence of FGC in the rural areas where poverty and lack of education are common, makes the religious leaders having a major say in banning the practice. Asking the advice of religious leaders of FGC is common in other settings such as Somalia [35].

Most participants indicated that they had received people complaining of FGC particularly husbands complaining of loss of sexual desire of their wives. Imams and preachers are not like health professionals to be in direct contact with the victims of FGC, but they come into indirect contact with some social problems resulting from FGC particularly through their role in marriage, divorce or when mediating over social problems. This highlights the important role they have in the community as they become indirectly exposed to the problems in the society. Mentioning such experience in this field also highlights the long-term social and psychological problems that result from FGC that lead to damaging the woman’s life.

The participants did not support having a law to ban FGC either because it will be against the *sharia* and religious advice on FGC or because they thought it would not work. While there is a need for strong laws and their enforcement to prevent people subjecting their daughters to the practice [36], lack of knowledge and awareness about these laws has remained a major concern [37, 38]. Many people think that raising the awareness of the people and actively involving religious leaders in combating FGC is more important than issuing or enforcing legislation. It is very important to have and enforce a law for combating FGM and prosecuting FGM practitioners and people who subject their daughters to the practice. However, legislation alone cannot end a harmful practice that is falsely linked to the religion and is embedded in the culture and traditions. There is a need for adopting proper mechanisms for enforcing such laws and raising the awareness of the people about their existence. In religious societies, it is important to have any law supported by the religion so that people accept and follow it.

## Limitations

This study is limited to Erbil governorate. However, some participants were originally from different areas of the Iraqi Kurdistan Region or had working experience outside Erbil governorate, and they comprehensively talked about their experience in these areas. Therefore, the results can partly provide an idea about the FGC problem in the Iraqi Kurdistan Region in general.

## Conclusions

The local religious leaders lack adequate knowledge about different aspects of FGC particularly the health consequences. There are different and disputing viewpoints about the reasons for practicing FGC, and there is poor support for having a law banning the practice. The different sectors of the government and the society, including the local religious leaders, need to take a strong stance against this unacceptable practice of FGC that is considered a severe violation of human rights. They also need to initiate vigorous action to stop this practice and protect young girls and women from its severe physical, psychosocial and reproductive consequences. There is an essential need for enhancing the knowledge of the local religious leaders regarding FGC and its adverse effects on the women’s health to motivate them to take a leading role in advising the people about this harmful practice. The local religious scholars and the Ministry of Endowments and Religious Affairs need to provide a clear message with clear evidence to the local religious leaders about FGC and the view of the Islamic religion on this practice. Topics on FGC could be integrated into the curriculum of the religious schools. Thus,

## Additional file

Additional file 1: Semi-structured questionnaire for assessing the knowledge and perspectives of religious leaders about female genital cutting. (DOCX 14 kb)

## Abbreviation

FGC: Female genital cutting

## Footnotes

1 A *fatwa* in the Islamic faith is a non-binding but authoritative legal opinion or learned interpretation that a qualified jurist could give on issues about the Islamic law

2 *Haram* is any act that is forbidden by Allah (God).

3 Imam is an Islamic leadership position, and it is most commonly used as the title of a worship leader or prayer leader of a mosque and the Muslim community.

4 *Khateeb* is a person who delivers the sermon (*khutbah*) during the Friday prayer and Eid prayers. The *khateeb* is usually the imam, but the two roles can be played by different people.

5 *Fiqh* is Islamic jurisprudence, is the human understanding of the *sharia*.

6 *Fuqaha* (singular Faqih) is an Islamic jurist, an expert in *fiqh*, or Islamic jurisprudence and Islamic Law Contents.

7 *Sunnah* includes the specific words, habits, practices, and silent permissions (or disapprovals) of the Prophet Muhammad (peace be upon him).

8 *Khutba* serves as the primary formal occasion for public preaching in the Islamic tradition. Such sermons occur regularly at the noon congregation prayer on Friday.

9 *Wajib* or its synonym *fard* is one of the five types of rules into which *fiqh* categorizes acts of every Muslim. It denotes a religious duty commanded by Allah. The Hanafi doctrine, however, makes a distinction between *wajib* and *fard*, the latter being obligatory and the former merely necessary.
