# Supplementary material for: Knowledge and perspectives of female genital cutting among the local religious leaders in Erbil governorate, Iraqi Kurdistan region
Source: Reprod Health. 2018 Mar 7;15:44. doi: 10.1186/s12978-018-0459-x (PMC5842576; doi:10.1186/s12978-018-0459-x)
Supplement: Supplementary file 1 — Semi-structured questionnaire for assessing the knowledge and perspectives of religious leaders about female genital cutting. (DOCX 14 kb) [file 12978_2018_459_MOESM1_ESM.docx]

**Additional file 1.** Semi-structured questionnaire for assessing the knowledge and perspectives of religious leaders about female genital cutting

1. What is your understanding of female genital cutting?

- Definition and description
- How many types of female genital cutting are there?
- For whom it is performed, at which age, who performs it, who decides on performing it?
- What are the advantages and disadvantages of female genital cutting?
- Why female genital cutting is practiced?

1. Do people come to you for advice on female genital cutting?

- How many?
- Who (women or men, poor or rich, educated or not educated, etc.)
- What they ask about?

1. Do people come to you to complain about the consequences of doing or not doing female genital cutting?

- What types of problems related to doing it (complications) or not doing it?

1. Why the practice is very common in some areas of Kurdistan (Sulaimania and Erbil) and not in others (Duhok)?
2. Why the practice is common in some Muslim countries (Kurdistan, Egypt, Sudan) and not others (other parts of Iraq, Jordan)?
3. What do you think about banning female genital cutting by a law?

- Support or not, why?
